# Durvalumab and pembrolizumab in advanced biliary tract cancer: a reconstructed patient-level mimic head-to-head comparative analysis

**DOI:** 10.3389/fimmu.2024.1497415

**Published:** 2024-12-23

**Authors:** Bi-Cheng Wang, Bo-Hua Kuang, Guo-He Lin, Chen Fu

**Affiliations:** ^1^ Cancer Center, Union Hospital, Tongji Medical College, Huazhong University of Science and Technology, Wuhan, China; ^2^ Department of Oncology, the Second Affiliated Hospital of Anhui Medical University, Hefei, China; ^3^ Wuhan No.1 Hospital, Wuhan, China

**Keywords:** biliary tract cancer, durvalumab, pembrolizumab, chemotherapy, mimic head-to-head comparative analysis

## Abstract

**Background:**

The addition of durvalumab or pembrolizumab to gemcitabine and cisplatin (GP) has been approved to statistically improve survival outcomes in patients with advanced biliary tract cancer. However, since the survival time was only prolonged by about two months, doubts have been raised. In this analysis, we aimed to evaluate the efficacy of combining durvalumab or pembrolizumab with GP chemotherapy.

**Methods:**

Records were identified through a formal search of PubMed and Web of Science. The TOPAZ-1 and KEYNOTE-966 trials were definitively included. Patient-level overall survival (OS) and progression-free survival (PFS) data were reconstructed and analyzed using a one-stage approach.

**Results:**

The immunotherapy plus GP chemotherapy group showed superiority over the GP chemotherapy group (OS: HR 0.83, p < 0.001; PFS: HR 0.88, p = 0.009). The survival outcomes were similar between the durvalumab and pembrolizumab groups (OS: HR 1.02, p = 0.83; PFS: HR 0.95, p = 0.53). In the subgroup analysis, the gemcitabine-maintenance group significantly prolonged the OS compared to the gemcitabine-limited-to-8-cycles group (OS: HR 0.86, p = 0.007). Neither the durvalumab nor pembrolizumab groups statistically improved the OS compared to the gemcitabine-maintenance group. In contrast, the durvalumab and pembrolizumab groups significantly improved OS compared to the gemcitabine-limited-to-8-cycles group.

**Conclusions:**

Based on this mimic head-to-head analysis, we are convinced that durvalumab and pembrolizumab benefit patients with biliary tract cancer. However, despite the statistically significant differences, the moderate progress made in OS and PFS might still be considered inadequate. It is crucial for clinicians to identify the precise subgroup population that could benefit most from immunotherapy and develop more strategies for those who might not respond well to immunotherapy.

## Introduction

Since 2010, gemcitabine plus cisplatin has been the standard of care for patients with advanced biliary tract cancer, based on the results reported by the ABC-02 trial (1). Over the next decade, numerous trials were conducted to identify more effective regimens, such as S-1, oxaliplatin, capecitabine, irinotecan, and fluorouracil (2–7). However, increasing the median overall survival (OS) to over 12 months has proven to be challenging. Even worse, the 2-year survival rate remains extremely low for patients with advanced biliary tract cancer (8).

Significant efforts have been made in targeted therapy; however, the results have been unsatisfactory. The ABC-03 trial showed that adding cediranib to gemcitabine plus cisplatin chemotherapy (OS: 14.1 months; progression-free survival [PFS]: 8.0 months) failed to improve survival outcomes compared with gemcitabine plus cisplatin chemotherapy alone (OS: 11.9 months; PFS: 7.4 months) in advanced biliary tract cancer (9). Bevacizumab combined with gemcitabine and cisplatin did not significantly prolong survival time (OS: 10.2 months; PFS: 8.1 months) compared with historical controls in first-line treatment (10). In a phase I trial for advanced cholangiocarcinoma with EGFR overexpression, participants received afatinib plus gemcitabine and cisplatin, resulting in a 7.7-month OS and a 6.0-month PFS (11).

Fortunately, a significant advancement brought by the TOPAZ-1 trial in 2022 has cheered clinicians and patients (12). Patients treated with durvalumab (anti-PD-L1 therapy) plus gemcitabine and cisplatin achieved a median OS of 12.8 months and a median PFS of 7.2 months. Based on these results, the combination therapy of durvalumab, gemcitabine, and cisplatin has been recommended as the preferred regimen for advanced biliary tract cancer by the National Comprehensive Cancer Network (NCCN) guidelines (13).

Progress in immunotherapy for advanced biliary tract cancer continued in 2023. The KEYNOTE-966 trial demonstrated that the median OS (12.7 months vs. 10.9 months) and PFS (6.5 months vs. 5.6 months) were significantly improved by adding pembrolizumab (anti-PD-1 therapy) to gemcitabine and cisplatin (14). According to the data from the KEYNOTE-966 trial, the latest NCCN guideline for biliary tract cancer has suggested gemcitabine and cisplatin in combination with pembrolizumab as the first-line treatment for advanced patients.

More specifically, detailed information should not be overlooked. For instance, patients enrolled in the TOPAZ-1 trial received durvalumab plus gemcitabine and cisplatin for up to eight cycles, followed by durvalumab every four weeks until disease progression or unacceptable toxicity (12). However, participants in the KEYNOTE-966 trial were treated with pembrolizumab (limited to 35 cycles), gemcitabine (without a maximum), and cisplatin (limited to eight cycles) until disease progression or unacceptable toxicity (14). Subsequently, more questions may arise. Which is more effective, anti-PD-L1 therapy or anti-PD-1 therapy? Does gemcitabine-maintenance therapy contribute to the survival time? Is the less-than-two-month increase in survival time meaningful for patients with advanced biliary tract cancer?

In this study, we collected the Kaplan-Meier survival curves from the TOPAZ-1 and KEYNOTE-966 trials and survival curves for gemcitabine plus cisplatin from all published randomized clinical trials. Patient-level data were reconstructed to conduct mimic head-to-head comparisons among the groups. Through our analysis, we aimed to provide not only more extensive survival data but also deeper insights for future treatments of advanced biliary tract cancer.

## Methods

### Study selection

For this reconstructed patient-level comparative analysis, the TOPAZ-1 and KEYNOTE-966 trials were definitively included. A systematic search was conducted on PubMed and Web of Science from inception to June 5, 2024, for additional randomized data on gemcitabine plus cisplatin chemotherapy. Search terms included “biliary tract cancer or BTC or cholangiocarcinoma or gallbladder cancer”, “unresectable or metastatic or advanced”, “gemcitabine”, and “cisplatin”. Each record was screened by two authors (B.C.W and C.F) for the gemcitabine plus cisplatin arm reported in randomized clinical trials. This analysis was conducted following the Preferred Reporting Items for Systematic Reviews and Meta-Analyses (PRISMA) reporting guideline for individual patient data (IPD) (15).

### Quality assessment and extraction of reported Kaplan-Meier curves

The risk of bias among the trials was evaluated by B.C.W. and C.F. using the Cochrane Risk of Bias Tool (16). Disagreements were discussed and resolved by a third reviewer (G.H.L.). The OS and PFS Kaplan-Meier curves and the number at risk data from the durvalumab/pembrolizumab plus gemcitabine and cisplatin arm and the gemcitabine plus cisplatin arm were extracted from the eligible trials. Since the updated curves of the KEYNOTE-966 trial were not yet reported in a formal article, and the survival data reported in the TOPAZ-1 studies were almost identical, we decided to extract the curves from the initially reported TOPAZ-1 and KEYNOTE-966 trials.

### Reconstruction of patient-level data and mimic head-to-head comparative analyses

Patient-level data were reconstructed using methods described in Liu’s report (17). The quality of the retrieved patient-level data was assessed by inspecting the shape of the survival curves, survival outcomes, survival rates, and hazard ratios (HRs). Mimic head-to-head comparative analyses were conducted using the one-stage method as described in Yap’s research (18). Both OS and PFS were designated as primary endpoints. The Cox proportional hazards regression model was applied to compute the hazard ratios. R software (version 4.3.2) was used to conduct all analyses, running the “ggplot2”, “survival”, “survminer”, and “IPDfromKM” packages. A P value < 0.05 was considered statistically significant.

## Results

Through systematic searching, we collected 689 and 1,069 records from the PubMed and Web of Science databases, respectively. Twelve randomized clinical trials published from 2009 to 2023 were eligible, including the TOPAZ-1 and KEYNOTE-966 trials (1–7, 9, 12, 14, 19, 20). Six of the twelve trials were phase 3 studies, and three were double-blind. In the pembrolizumab plus gemcitabine and cisplatin group, patients were treated with pembrolizumab (200 mg on day 1), gemcitabine (1,000 mg/m^2^ on days 1 and 8), and cisplatin (25 mg/m^2^ on days 1 and 8) every 21 days. In the durvalumab plus gemcitabine and cisplatin group, patients were treated with durvalumab (1,500 mg on day 1), gemcitabine (1,000 mg/m^2^ on days 1 and 8), and cisplatin (25 mg/m^2^ on days 1 and 8) every 21 days. Although both groups received immunotherapy combined with chemotherapy, some differences existed. Pembrolizumab was administered for up to 35 cycles, while durvalumab was administered without a maximum limit. In the KEYNOTE-966 trial, there was no limit on the number of cycles of gemcitabine, but in the TOPAZ-1 trial, it was limited to 8 cycles. Considering the contribution of gemcitabine maintenance treatment to survival outcomes, we also collected survival data on gemcitabine and cisplatin in advanced biliary tract cancer reported in all randomized clinical trials. The basic characteristics of the included trials are listed in **Table 1**. Additionally, the disease characteristics, including gender, performance status, original tumor site, and disease stage, were generally comparable between the immune-chemotherapy group (pembrolizumab/durvalumab + gemcitabine + cisplatin) and the chemotherapy group (gemcitabine + cisplatin ± placebo) (**Table 2**).

**Table 1:** Basic characteristics of the enrolled trials.

Study	Trials identifier	Clinical trial name	Study phase	Study design	Immunotherapy regimen	Chemotherapy regimen
Robin Kate Kelley, 2023 (14)	NCT04003636	KEYNOTE-966	III	Double blind, randomized	Pembrolizumab: 200 mg, ivdrip, q3w, limited to 35 cycles	Gemcitabine: 1000 mg/m^2^, ivdrip, day 1 and day 8, q3w, without maximum Cisplatin: 25 mg/m^2^, ivdrip, day 1 and day 8, q3w, limited to 8 cycles
Tatsuya Ioka, 2023 (7)	NCT02182778	KHBO1401- MITSUBA	III	Open label, randomized	\	Gemcitabine: 1000 mg/m2, ivdrip, day 1 and day 8, q3w, limited to 8 cycles Cisplatin: 25 mg/m^2^, ivdrip, day 1 and day 8, q3w, limited to 8 cycles
Jean marc Phelip, 2022 (6)	NCT02591030	PRODIGE 38 AMEBICA	II	Open label, randomized	\	Gemcitabine: 1000 mg/m^2^, ivdrip, day 1 and day 8, q3w, limited to 8 cycles Cisplatin: 25 mg/m^2^, ivdrip, day 1 and day 8, q3w, limited to 8 cycles
Do-Youn Oh, 2022 (12)	NCT03875235	TOPAZ-1	III	Double blind, randomized	Durvalumab: 1500 mg, ivdrip, q3w, limited to 8 cycles; then 1500 mg, ivdrip, q4w, maintenance	Gemcitabine: 1000 mg/m^2^, ivdrip, day 1 and day 8, q3w, limited to 8 cycles Cisplatin: 25 mg/m^2^, ivdrip, day 1 and day 8, q3w, limited to 8 cycles
Alice Markussen, 2020 (5)	2013-004854-46	\	II	Open label, randomized	\	Gemcitabine: 1000 mg/m^2^, ivdrip, day 1 and day 8, q3w, limited to 8 cycles Cisplatin: 25 mg/m^2^, ivdrip, day 1 and day 8, q3w, limited to 8 cycles
Atul Sharma, 2019 (4)	CTRI/2010/091/001406	\	III	Open label, randomized	\	Gemcitabine: 1000 mg/m^2^, ivdrip, day 1 and day 8, q3w, limited to 8 cycles Cisplatin: 25 mg/m^2^, ivdrip, day 1 and day 8, q3w, limited to 8 cycles
Chigusa Morizane, 2019 (3)	UMIN00001066	JCOG1113	III	Open label, randomized	\	Gemcitabine: 1000 mg/m2, ivdrip, day 1 and day 8, q3w, limited to 8 cycles; then 1000 mg/m2, ivdrip, day 1, day 8, and day 15, without maximum Cisplatin: 25 mg/m2, ivdrip, day 1 and day 8, q3w, limited to 8 cycles
Juan Valle, 2015 (9)	NCT00939848	ABC-03	II	Double blind, randomized	\	Gemcitabine: 1000 mg/m2, ivdrip, day 1 and day 8, q3w, limited to 8 cycles Cisplatin: 25 mg/m2, ivdrip, day 1 and day 8, q3w, limited to 8 cycles
Myoung Joo Kang, 2012 (2)	NCT 01375972	\	II	Open label, randomized	\	Gemcitabine: 1000 mg/m2, ivdrip, day 1 and day 8, q3w, limited to 6 cycles; then 1000 mg/m2, ivdrip, day 1 and day 8, without maximum Cisplatin: 60 mg/m2, ivdrip, day 1, q3w, limited to 6 cycles
Juan Valle, 2010 (1)	NCT00262769	ABC-02	III	Open label, randomized	\	Gemcitabine: 1000 mg/m2, ivdrip, day 1 and day 8, q3w, limited to 8 cycles Cisplatin: 25 mg/m2, ivdrip, day 1 and day 8, q3w, limited to 8 cycles
Takuji Okusaka, 2010 (20)	\	\	II	Open label, randomized	\	Gemcitabine: 1000 mg/m2, ivdrip, day 1 and day 8, q3w, limited to 16 cycles Cisplatin: 25 mg/m2, ivdrip, day 1 and day 8, q3w, limited to 16 cycles
Juan Valle, 2009 (19)	\	ABC-01	II	Open label, randomized	\	Gemcitabine: 1000 mg/m2, ivdrip, day 1 and day 8, q3w, limited to 8 cycles Cisplatin: 25 mg/m2, ivdrip, day 1 and day 8, q3w, limited to 8 cycles

**Table 2:** Basic characteristics of the enrolled patients.

Group	DUR/PEM+GP		GP2														
Subgroup	PEM+GP	DUR+GP	DUR/PEM+GP	GP1												GP1	GP2
Study	Robin Kate Kelley, 2023 (14)	Do-Youn Oh, 2022 (12)		Robin Kate Kelley, 2023 (14)	Do-Youn Oh, 2022 (12)	Tatsuya Ioka, 2023 (7)	Jean marc Phelip, 2022 (6)	Alice Markussen, 2020 (5)	Atul Sharma, 2019 (4)	Chigusa Morizane, 2019 (3)	Juan Valle, 2015 (9)	Myoung Joo Kang, 2012 (2)	Juan Valle, 2010 (1)	Takuji Okusaka, 2010 (20)	Juan Valle, 2009 (19)		
No. patients	533	341	874	536	344	123	96	49	124	175	62	49	204	41	42	880	1845
Age, years	64.0 (IQR 57.0-71.0)	64 (range 20-84)		63.0 (IQR 55.0-70.0)	64 (range 31-85)	68 (range 40-84)	63 (IQR 55-67)	65 (range 39-82)	Mean 47.8 (SD ± 12)	67 (range 41-78)	64.5 (IQR 59.7-73.1)	59 (range 32-77)	63.9 (range 32.8-81.9)	65 (range 43-80)	63 (range 38-76)		
gender - No. (%)																	
male	280 (52.5)	169 (49.6)	449 (51.4)	272 (50.7)	176 (51.2)	66 (53.7)	47 (49.0)	23 (46.9)	39 (31.5)	99 (56.6)	28 (45.2)	31 (63.3)	96 (47.1)	18 (43.9)	17 (40.5)	448 (50.9)	912 (49.4)
female	253 (47.5)	172 (50.4)	425 (48.6)	264 (49.3)	168 (48.8)	57 (46.3)	49 (51.0)	26 (53.1)	85 (68.5)	76 (43.4)	34 (54.8)	18 (36.7)	108 (52.9)	23 (56.1)	25 (59.5)	432 (49.1)	933 (50.6)
ECOG performance status of 0 - No. (%)	258 (48.4)	173 (50.7)	431 (49.3)	228 (42.5)	163 (47.4)	121 (98.4)	46 (47.9)	23 (46.9)	7 (5.6)	130 (74.3)	28 (45.2)		66 (32.4)	34 (82.9)	5 (11.9)	391 (44.4)	851 (47.4)
Primary tumor site - No. (%)																	
Intrahepatic	320 (60.0)	190 (55.7)	510 (58.4)	313 (58.4)	193 (56.1)	43 (35.0)	59 (61.5)	31 (63.3)		50 (28.6)	15 (24.2)	20 (40.8)	131 (64.2)	14 (34.1)	12 (28.6)	506 (57.5)	750 (49.4)
Extrahepatic (ampullary)	98 (18.4)	66 (19.4)	164 (18.8)	105 (19.6)	65 (18.9)	40 (32.5)	20 (20.8)	5 (10.2)		56 (32.0)	28 (45.2)	16 (32.7)		12 (29.3)	10 (23.8)	170 (19.3)	357 (23.5)
Gallbladder	115 (21.6)	85 (24.9)	200 (22.9)	118 (22.0)	86 (25.0)	40 (32.5)	17 (17.7)	11 (22.4)		68 (38.9)	19 (30.6)	13 (26.5)	73 (35.8)	15 (36.6)	10 (23.8)	204 (23.2)	470 (27.3)
Disease stage																	
Locally advanced	60 (11.3)	38 (11.1)	98 (11.2)	66 (12.3)	57 (16.6)	32 (26.0)	13 (13.5)	12 (24.5)		31 (17.7)	8 (12.9)	14 (28.6)	55 (27.0)	30 (73.2)	16 (38.1)	123 (14.0)	334 (19.4)
Metastatic (recurrent)	473 (88.7)	303 (88.9)	776 (88.8)	470 (87.7)	286 (83.1)	91 (74.0)	83 (86.5)	36 (73.5)		142 (81.1)	54 (87.1)	35 (71.4)	149 (73.0)	11 (26.8)	26 (61.9)	756 (85.9)	1383 (80.4)

DUR, durvalumab; PEM, pembrolizumab; GP, gemcitabine + cisplatin; GP1, gemcitabine + cisplatin in the TOPAZ-1 and KEYNOTE-966 trials; GP2, gemcitabine + cisplatin in all enrolled trials; IQR, interquartile range; SD, standard deviation.

**Figure 1:**
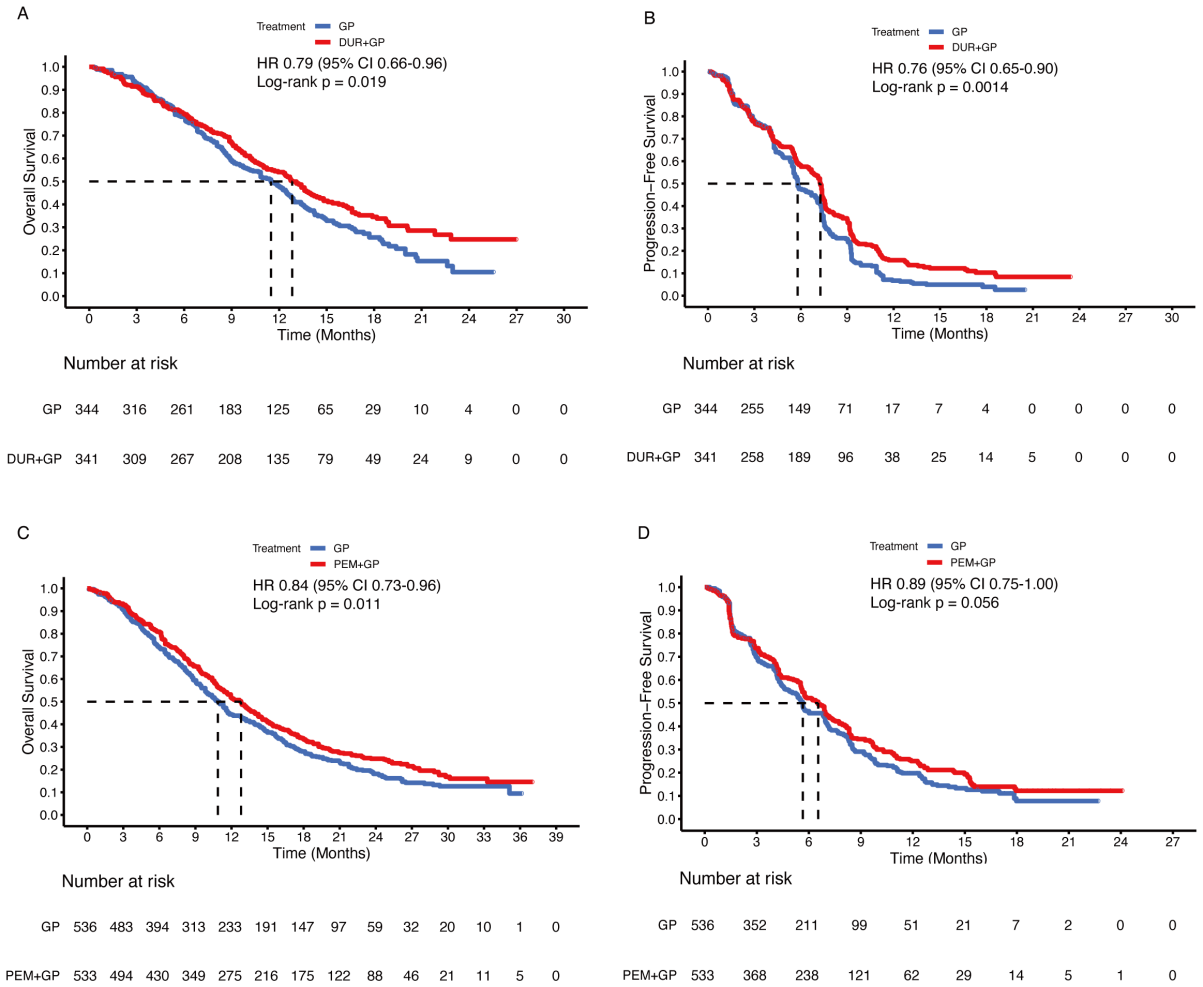
Reconstructed Kaplan-Meier curves for overall and progression-free survival of the TOPAZ-1 **(A, B)** and KEYNOTE-966 **(C, D)** trials.

Before conducting the mimic head-to-head comparisons, the patient-level data from the TOPAZ-1 and KEYNOTE-966 trials were reconstructed to confirm the feasibility of the methods used in this analysis. For durvalumab, the reconstructed HRs were 0.79 (95% CI 0.66-0.96; p = 0.019) for OS and 0.76 (95% CI 0.65-0.90; p = 0.001) for PFS (original: OS 0.75, 95% CI 0.63-0.89; p = 0.001, PFS 0.80, 95% CI 0.66-0.97; p = 0.021) (**Figures 1A, B**). The median OS was 12.8 months (95% CI, 11.2-14.2) in the durvalumab arm versus 11.4 months (95% CI, 10.1-12.6) in the placebo arm (original: 12.8 months [95% CI, 11.1-14.0] vs. 11.5 months [95% CI, 10.1-12.5]). The median PFS was 7.2 months (95% CI, 6.7-7.4) in the durvalumab arm compared to 5.8 months (95% CI, 5.5-6.7) in the placebo arm (original: 7.2 months [95% CI, 6.7-7.4] versus 5.7 months [95% CI, 5.6-6.7]). For pembrolizumab, the reconstructed HRs were 0.84 (95% CI 0.73-0.96; p = 0.011) for OS and 0.89 (95% CI 0.75-1.00; p = 0.056) for PFS (original: OS 0.83, 95% CI 0.72-0.95; p = 0.003, PFS 0.86, 95% CI 0.75-1.00; p = 0.023) (**Figures 1C, D**). The median OS was 12.7 months (95% CI 11.5-13.6) in the pembrolizumab arm vs. 10.8 months (95% CI 9.9-11.7) in the placebo arm (original: 12.7 months [95% CI 11.5-13.6] vs. 10.9 months [95% CI 9.9-11.6]). The median PFS was 6.5 months [95% CI 5.7-6.9] in the pembrolizumab arm versus 5.6 months [95% CI 5.1-6.8] in the placebo arm (original: 6.5 months [95% CI 5.7-6.9] vs. 5.6 months [95% CI 5.1-6.6]). Based on the results, our reconstructed Kaplan-Meier survival curves and Cox regression analyses demonstrated satisfactory repeatability compared with the originally published curves and data. Subsequently, patient-level data on gemcitabine plus cisplatin were reconstructed based on other enrolled randomized clinical trials.

### Mimic head-to-head comparisons

**Figure 2:**
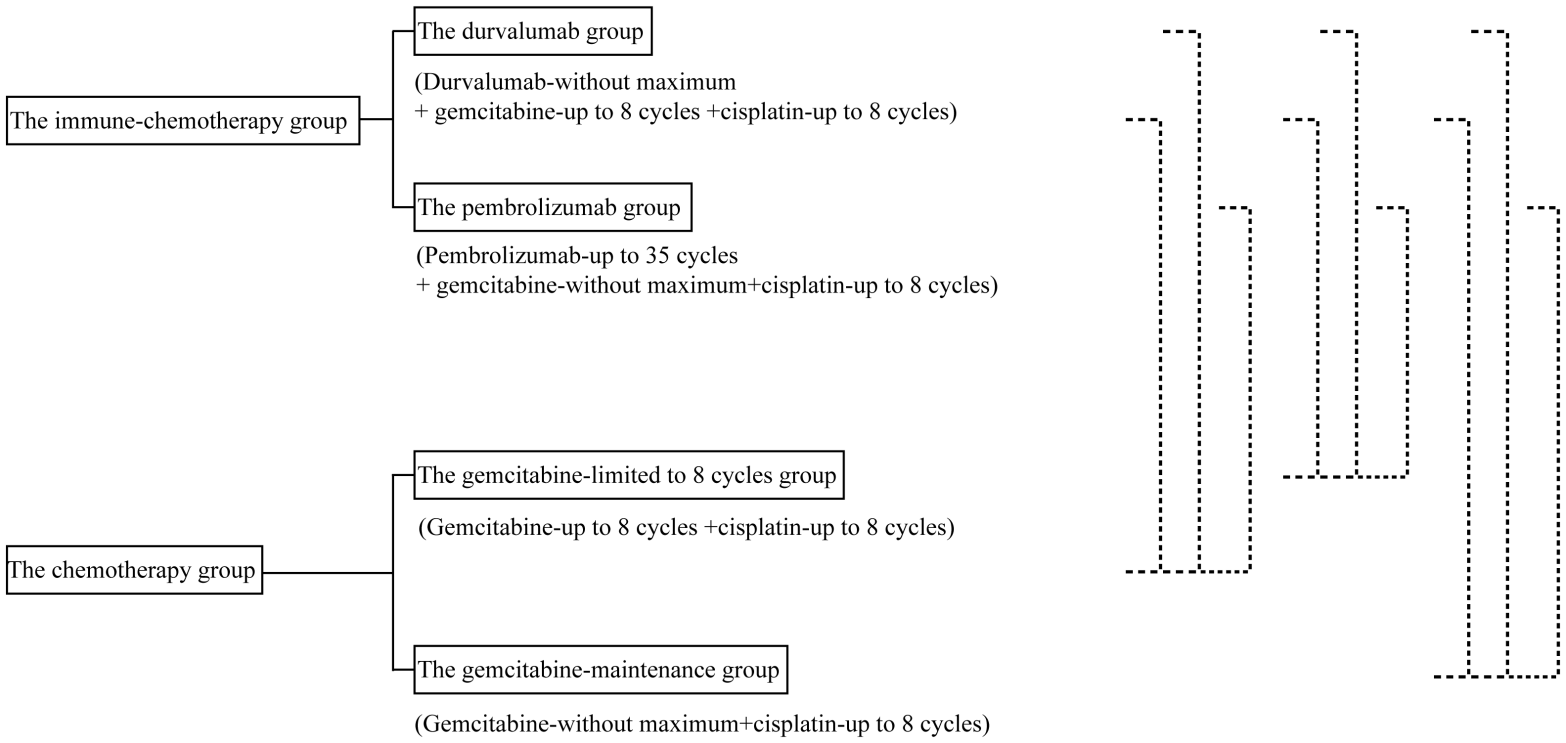
The design of mimic head-to-head comparisons.

**Figure 2** shows the design of the mimic head-to-head comparisons. The survival data for Myoung Joo Kang’s trial were not reconstructed due to the absence of the number-at-risk data.

**Figure 3:**
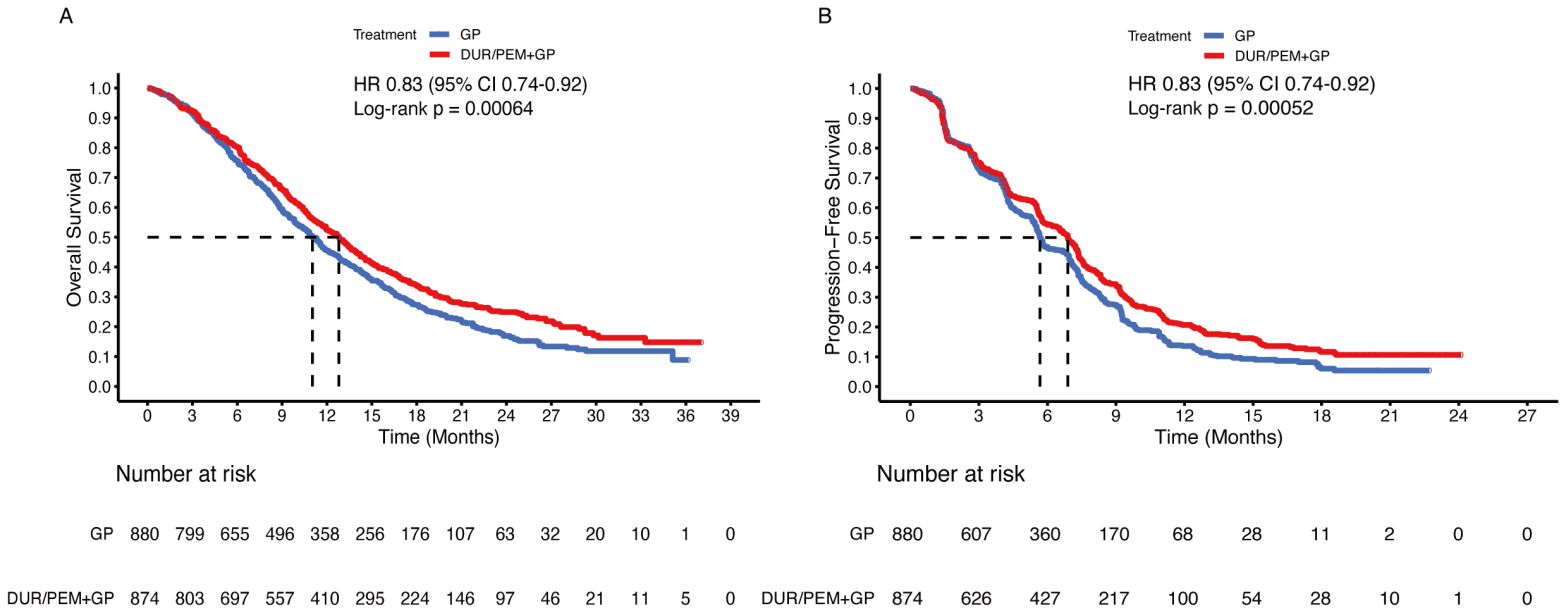
Pooled Kaplan-Meier curves of overall and progression-free survival comparing gemcitabine and cisplatin plus durvalumab/pembrolizumab (red) with gemcitabine and cisplatin (blue). Kaplan-Meier curves are presented for overall survival **(A)** and progression-free survival **(B)**. Dur+Pem denotes gemcitabine + cisplatin + durvalumab or pembrolizumab, and Placebo-1 denotes gemcitabine + cisplatin (Data were reconstructed from the TOPAZ-1 and the KEYNOTE-966 trials).

By synthesizing the reconstructed patient-level data of the TOPAZ-1 and KEYNOTE-966 trials, we found that the median OS was 12.8 months (95% CI 11.9-13.5) in the immunotherapy plus chemotherapy group and 11.0 months (95% CI 10.3-11.7) in the placebo plus chemotherapy group (HR 0.83, 95% CI 0.74-0.92; p < 0.001) (**Figure 3A**). The median PFS was 6.9 months (95% CI 6.4-7.2) in the immunotherapy plus chemotherapy group and 5.7 months (95% CI 5.5-5.9) in the placebo plus chemotherapy group (HR 0.83, 95% CI 0.74-0.92; p < 0.001) (**Figure 3B**).

**Figure 4:**
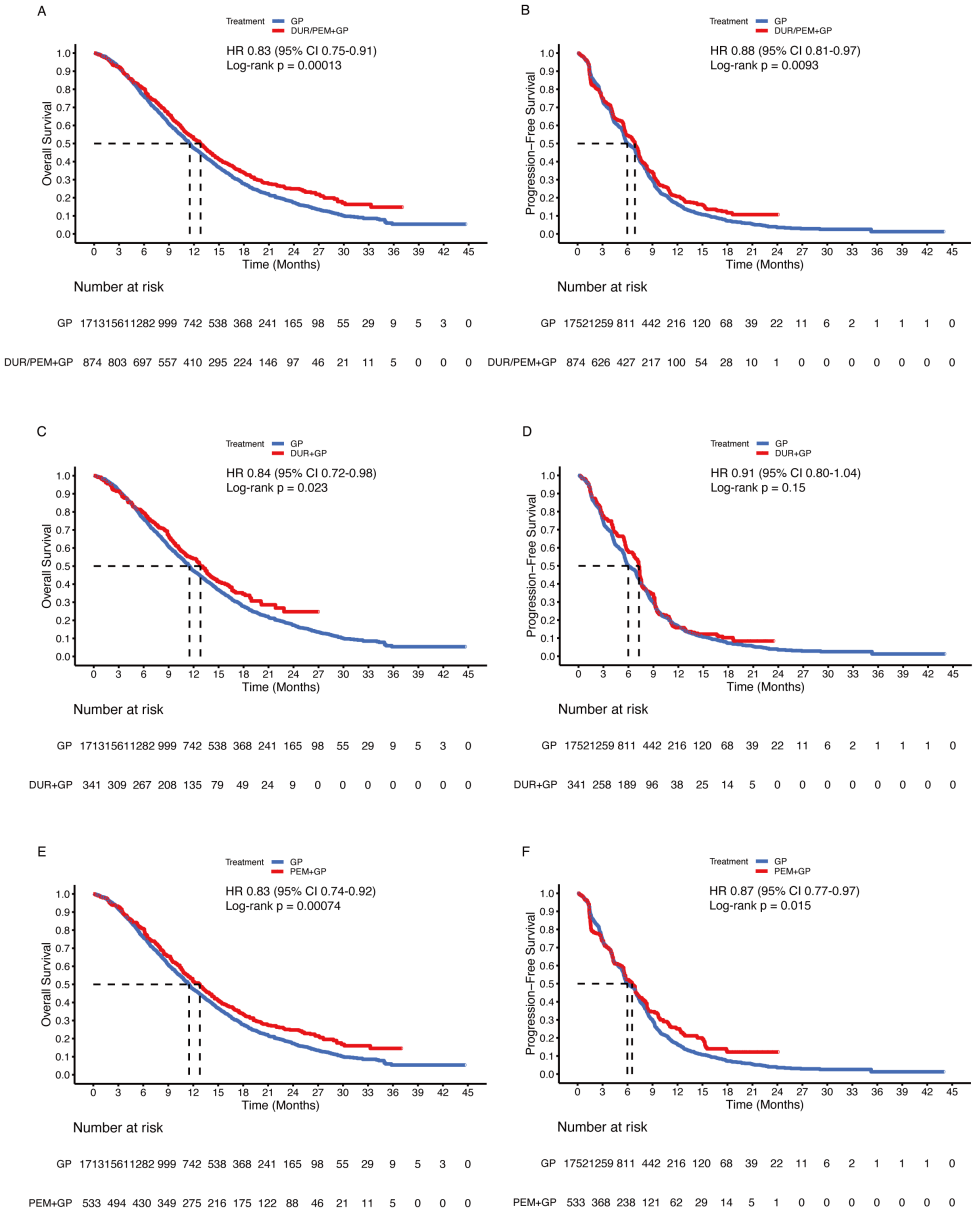
Pooled Kaplan-Meier curves of overall (left panels) and progression-free (right panels) survival comparing gemcitabine and cisplatin plus durvalumab/pembrolizumab with gemcitabine and cisplatin. Gemcitabine and cisplatin combined with durvalumab or pembrolizumab versus gemcitabine and cisplatin **(A, B)**. Gemcitabine and cisplatin combined with durvalumab (red) versus gemcitabine and cisplatin (blue) **(C, D)**. Gemcitabine and cisplatin combined with pembrolizumab (red) versus gemcitabine and cisplatin (blue) **(E, F)**. Dur+Pem denotes durvalumab/pembrolizumab + gemcitabine + cisplatin, Dur durvalumab + gemcitabine + cisplatin, Pem pembrolizumab + gemcitabine + cisplatin, and Placebo-2 gemcitabine + cisplatin (Data were reconstructed from all enrolled trials).

**Figure 5:**
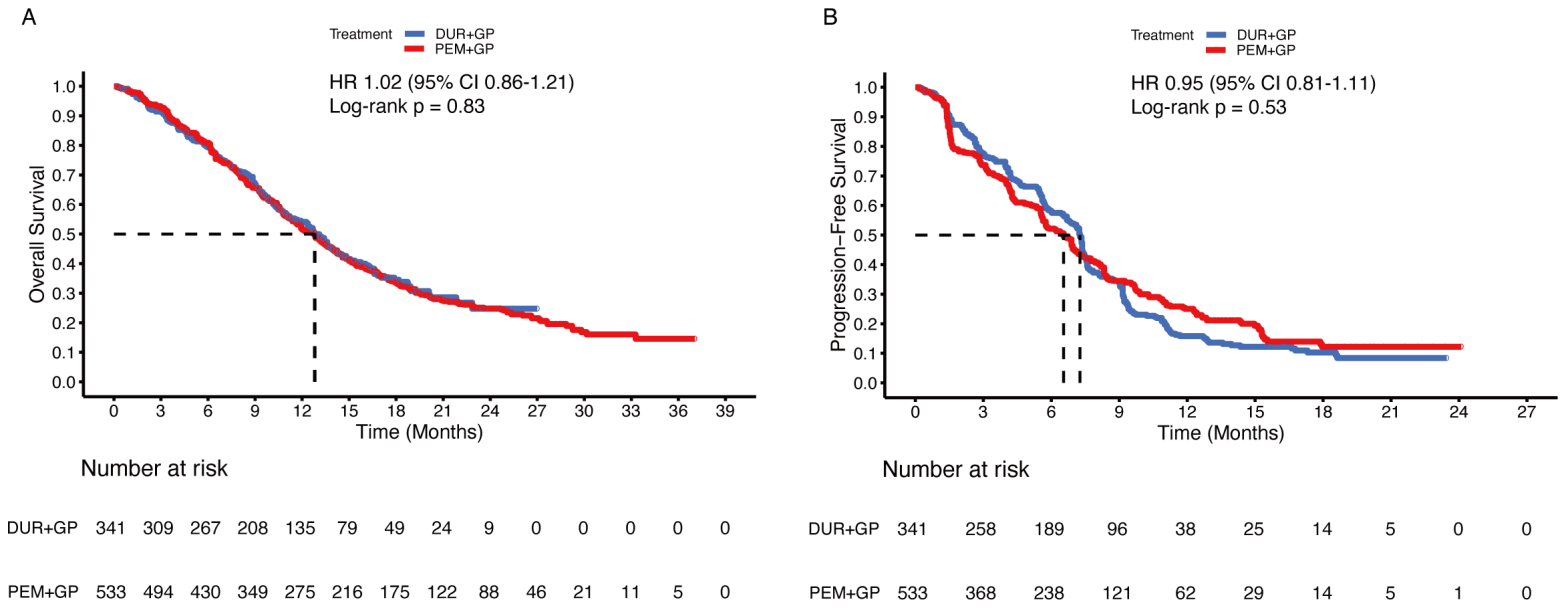
Pooled Kaplan-Meier curves of overall and progression-free survival comparing durvalumab (blue) with pembrolizumab (red) in combination with gemcitabine and cisplatin. Kaplan-Meier curves are presented for overall survival **(A)** and progression-free survival **(B)**. Durvalumab denotes durvalumab + gemcitabine + cisplatin, and Pembrolizumab denotes pembrolizumab + gemcitabine + cisplatin.

Comparing the immune-chemotherapy group (durvalumab/pembrolizumab + gemcitabine + cisplatin) with the chemotherapy group (gemcitabine + cisplatin ± placebo), HR was 0.83 (95% CI 0.75-0.91; p < 0.001) for OS and 0.88 (95% CI 0.81-0.97; p = 0.009) for PFS (**Figures 4A, B**). The median OS and PFS in the chemotherapy group were 11.5 months (95% CI 10.9-11.9) and 6.0 months (95% CI 5.7-6.8), respectively. In the subgroup analysis, the durvalumab and pembrolizumab groups showed statistically significant improvement in OS (**Figures 4C, E**). For PFS, the difference between the durvalumab group (7.2 months) and the chemotherapy group (6.0 months) was not statistically significant (HR 0.91, 95% CI 0.80-1.04; p = 0.15), while the pembrolizumab group (6.5 months) was statistically superior to the chemotherapy group (6.0 months) (**Figures 4D, F**). Comparing the durvalumab group with the pembrolizumab group, the HR was 1.02 (95% CI 0.86-1.21; p = 0.83) for OS and 0.95 (95% CI 0.81-1.11; p = 0.53) for PFS (**Figure 5**).

**Figure 6:**
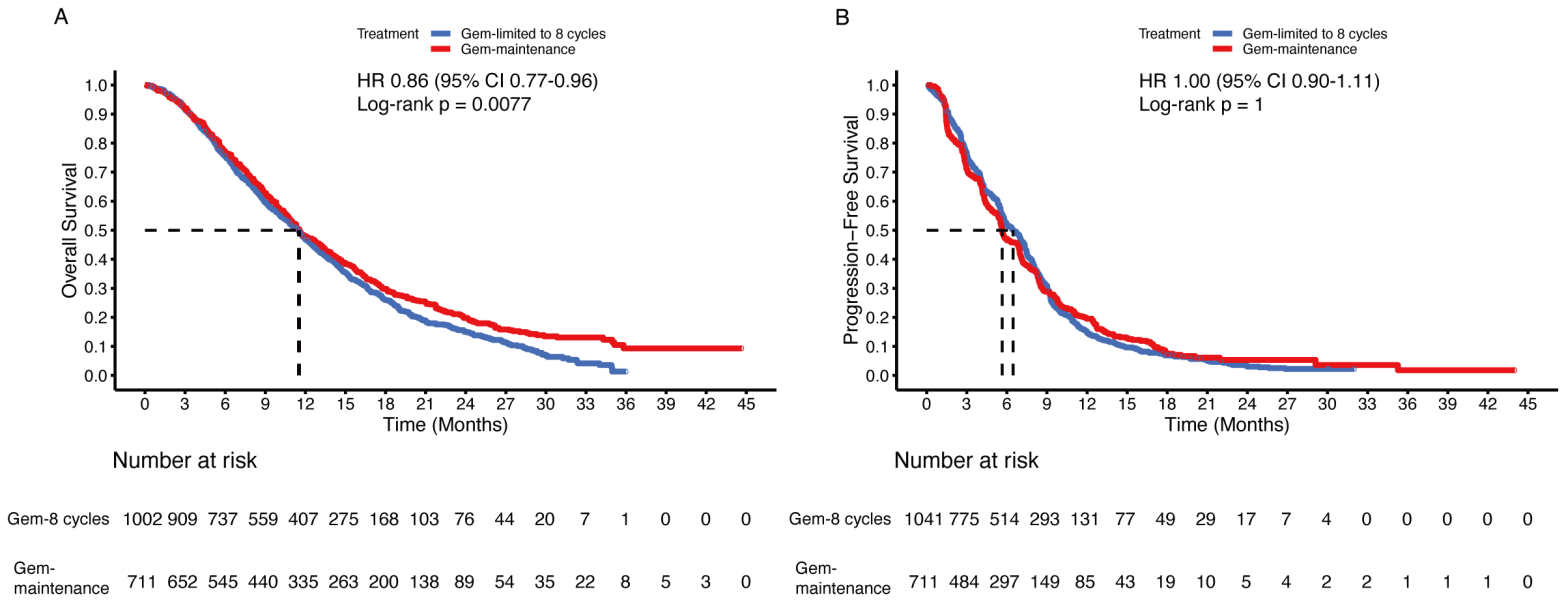
Pooled Kaplan-Meier curves of overall and progression-free survival comparing gemcitabine limited to 8 cycles plus cisplatin (blue) with gemcitabine without maximum (red) plus cisplatin combined with cisplatin. Kaplan-Meier curves are presented for overall survival **(A)** and progression-free survival **(B)**. Gem-limited to 8 cycles denotes gemcitabine limited to 8 cycles plus cisplatin, and Gem-maintenance denotes gemcitabine without maximum plus cisplatin.

To determine whether patients could benefit from gemcitabine maintenance treatment, the chemotherapy group was divided into two subgroups: the gemcitabine-limited-to-8-cycles group and the gemcitabine-maintenance group. After the mimic head-to-head comparison, we found that the gemcitabine maintenance group showed a statistically significant improvement in OS (HR 0.86, 95% CI 0.77-0.96; p = 0.008) compared to the gemcitabine limited to 8 cycles group, but sharing same median OS (11.5 months [95% CI 10.9-12.7] vs. 11.5 months [95% CI 10.7-12.0]) (**Figure 6A**). Additionally, no statistically significant differences were observed between the groups regarding PFS (HR 1.00, 95% CI 0.90-1.11; p = 1), even the median PFS was 5.6 months (95% CI 5.5-6.3) in the gemcitabine maintenance group versus 6.5 months (95% CI 5.8-7.0) in the gemcitabine limited to 8 cycles group (**Figure 6B**).

**Figure 7:**
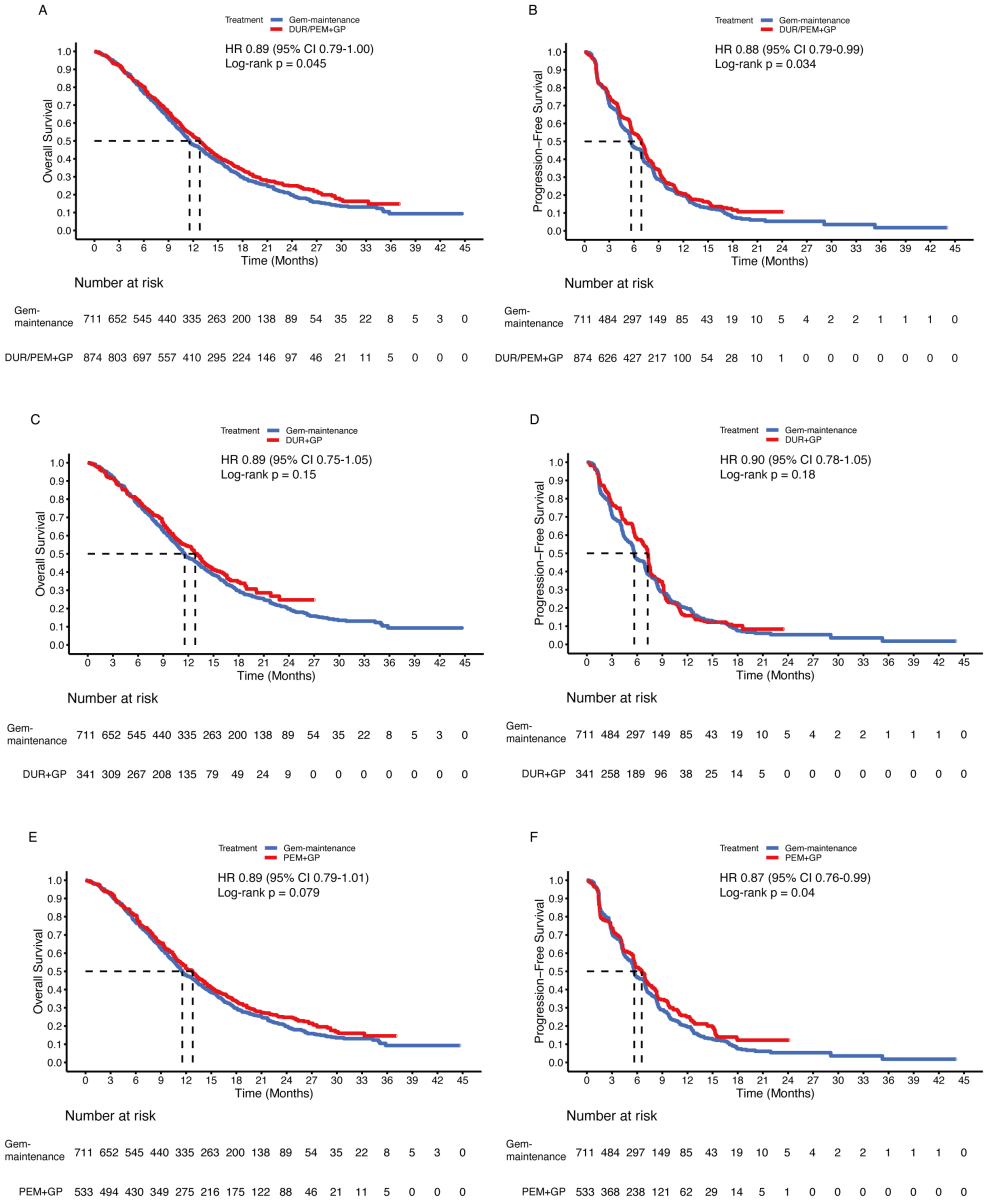
Pooled Kaplan-Meier curves of overall (left panels) and progression-free (right panels) survival comparing gemcitabine and cisplatin plus durvalumab/pembrolizumab with gemcitabine without maximum and cisplatin. Gemcitabine and cisplatin combined with durvalumab or pembrolizumab versus gemcitabine without maximum and cisplatin **(A, B)**. Gemcitabine and cisplatin combined with durvalumab (red) versus gemcitabine without maximum and cisplatin (blue) **(C, D)**. Gemcitabine and cisplatin combined with pembrolizumab (red) versus gemcitabine without maximum and cisplatin (blue) **(E, F)**. Gem-maintenance denotes gemcitabine without maximum plus cisplatin, Durvalumab denotes durvalumab + gemcitabine + cisplatin, and Pembrolizumab denotes pembrolizumab + gemcitabine + cisplatin.

Since gemcitabine maintenance treatment contributed to OS, whether the addition of pembrolizumab further prolonged survival time is an important question. In the comparison between the immunotherapy plus chemotherapy group and the gemcitabine maintenance group, the HR was 0.89 (95% CI 0.79-1.00; p = 0.045) for OS and 0.88 (95% CI 0.79-0.98; p = 0.034) for PFS (**Figures 7A, B**). In subgroup analysis, the durvalumab group (up to 8 cycles of chemotherapy) did not statistically improve OS (HR 0.89, 95% CI 0.75-1.05; p = 0.15) or PFS (HR 0.90, 95% CI 0.78-1.05; p = 0.18) compared to the gemcitabine maintenance group (**Figures 7C, D**). Despite the significant improvement in PFS (HR 0.87, 95% CI 0.76-0.99; p = 0.04), there was no significant difference in OS between the pembrolizumab group (without maximum gemcitabine) and the gemcitabine maintenance group (HR 0.89, 95% CI 0.79-1.01; p = 0.079) (**Figures 7E, F**).

**Figure 8:**
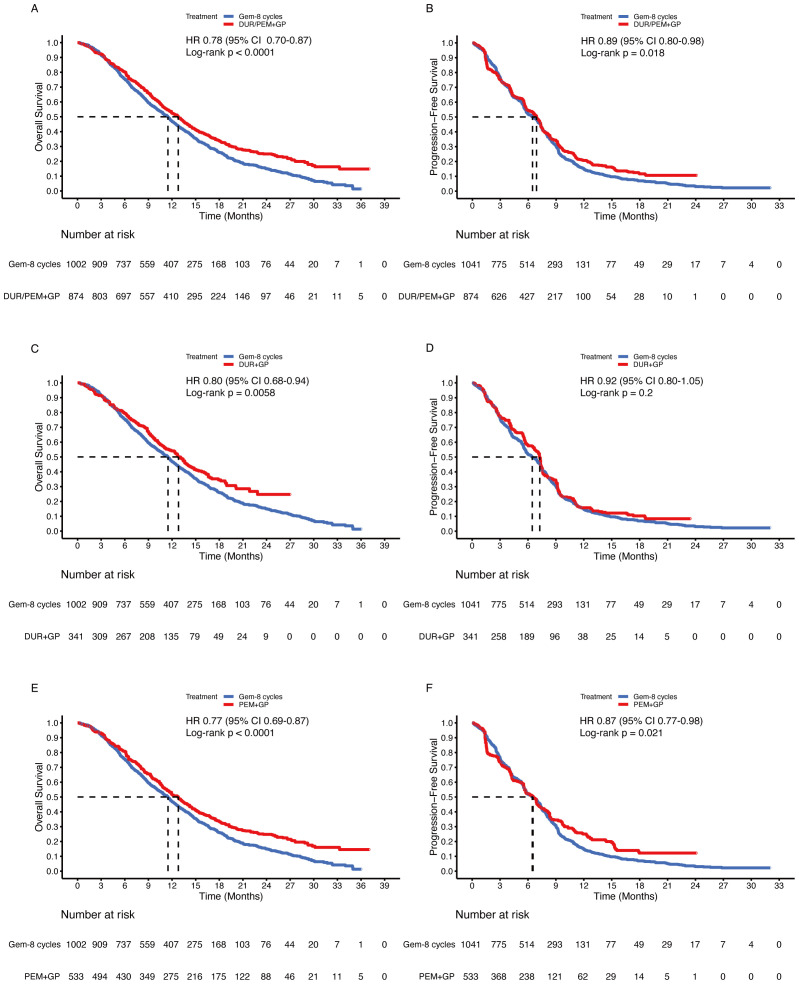
Pooled Kaplan-Meier curves of overall (left panels) and progression-free (right panels) survival comparing gemcitabine and cisplatin plus durvalumab/pembrolizumab with gemcitabine limited to 8 cycles and cisplatin. Gemcitabine and cisplatin combined with durvalumab or pembrolizumab versus gemcitabine limited to 8 cycles and cisplatin **(A, B)**. Gemcitabine and cisplatin combined with durvalumab (red) versus gemcitabine limited to 8 cycles and cisplatin (blue) **(C, D)**. Gemcitabine and cisplatin combined with pembrolizumab (red) versus gemcitabine limited to 8 cycles and cisplatin (blue) **(E, F)**. Gem-maintenance denotes gemcitabine without maximum plus cisplatin, Durvalumab denotes durvalumab + gemcitabine + cisplatin, and Pembrolizumab denotes pembrolizumab + gemcitabine + cisplatin.

To confirm the benefits of the gemcitabine-maintenance treatment, the gemcitabine limited to 8 cycles group was compared. The immune-chemotherapy group was statistically superior to the gemcitabine-limited-to-8-cycles group in terms of OS (HR 0.78, 95% CI 0.70-0.87; p < 0.001) and PFS (HR 0.89, 95% CI 0.80-0.98; p = 0.018) (**Figures 8A, B**). In subgroup analysis, the durvalumab group statistically improved OS (HR 0.80, 95% CI 0.68-0.94; p = 0.005) but not PFS (HR 0.92, 95% CI 0.80-1.05; p = 0.2) compared to the gemcitabine maintenance group (**Figures 8C, D**). For pembrolizumab, the OS (HR 0.77, 95% CI 0.69-0.87; p < 0.001) and PFS (HR 0.87, 95% 0.77-0.98; p = 0.021) were significantly improved (**Figures 8E, F**).

For survival rates, the 6-month PFS rate was 54.5% (95% CI 51.2-58.1) in the immune-chemotherapy group and 50% (95% CI 47.6-52.4) in the chemotherapy group. The 12-month OS rate was 52.6% (95% CI 49.4-56.1) in the immune-chemotherapy group and 47.3% (95% CI 44.9-49.8) in the chemotherapy group. In **Table 3**, we summarize the reconstructed median survival times and survival rates to provide detailed information for each group.

**Table 3:** Reconstructed median survival outcomes.

Groups	DUR+GP	PEM+GP	DUR/PEM+GP	GP1	GP2	Gem-limited to 8 cycles	Gem-maintenance
Overall survival							
Median (months)	12.8 (11.2-14.2)	12.7 (11.5-13.6)	12.8 (11.9-13.5)	11.0 (10.3-11.7)	11.5 (10.9-11.9)	11.5 (10.7-12.0)	11.5 (10.9-12.)
6-month (%, 95% CI)	79.6 (75.4-84.0)	80.9 (77.6-84.3)	80.3 (77.8-83.0)	75.7 (72.9-78.6)	76.2 (74.2-78.2)	75.4 (72.8-78.2)	77.1 (74.1-80.2)
12-month (%, 95% CI)	54.1 (48.8-59.9)	51.9 (47.8-56.3)	52.6 (49.4-56.1)	45.6 (42.4-49.1)	47.3 (44.9-49.8)	46.8 (43.7-50.1)	48.0 (44.5-51.9)
18-month (%, 95% CI)	34.5 (28.9-41.2)	33.5 (29.7-37.8)	33.8 (30.6-37.3)	27.4 (24.4-30.7)	27.8 (25.6-30.1)	26.1 (23.2-29.3)	29.8 (26.6-33.4)
24-month (%, 95% CI)	24.7 (18.2-33.5)	24.8 (21.3-28.9)	24.9 (21.9-28.5)	16.9 (14.1-20.2)	17.3 (15.4-19.5)	15.0 (12.5-18.0)	19.8 (16.9-23.2)
36-month (%, 95% CI)		14.6 (10.5-20.2)	14.8 (10.8-20.3)	8.9 (4.8-16.6)	5.4 (3.5-8.2)	1.3 (0.2-6.8)	9.3 (6.1-14.1)
Progression-free survival							
Median (months)	7.2 (6.7-7.4)	6.5 (5.7-6.9)	6.9 (6.4-7.2)	5.7 (5.5-5.9)	6 (5.7-6.8)	6.5 (5.8-7.0)	5.6 (5.5-6.3)
6-month (%, 95% CI)	58.1 (53.1-63.7)	52.1 (47.9-56.8)	54.5 (51.2-58.1)	46.9 (43.5-50.4)	50.0 (47.6-52.4)	51.9 (48.9-55.0)	47.1 (43.5-51.1)
12-month (%, 95% CI)	15.8 (12.1-20.7)	25.0 (21.0-29.8)	20.7 (17.8-24.1)	13.6 (11.2-16.6)	16.4 (14.6-18.4)	14.8 (12.7-17.3)	19.5 (16.5-23.2)
18-month (%, 95% CI)	10.3 (7.0-15.0)	12.2 (8.4-17.7)	11.7 (9.0-15.1)	6.0 (3.9-9.2)	7.2 (5.8-8.8)	6.9 (5.4-8.9)	7.5 (5.2-10.8)
24-month (%, 95% CI)		12.2 (8.4-17.7)	10.7 (8.0-14.2)		3.5 (2.5-5.0)	3.0 (2.0-4.7)	5.4 (3.2-8.9)
36-month (%, 95% CI)					1.3 (0.2-5.5)		1.8 (0.3-9.6)

DUR, durvalumab; PEM, pembrolizumab; GP, gemcitabine + cisplatin; GP1, gemcitabine + cisplatin in the TOPAZ-1 and KEYNOTE-966 trials; GP2, gemcitabine + cisplatin in all enrolled trials; Gem, gemcitabine.

## Discussion

In this mimic head-to-head comparative analysis, our results demonstrated that immunotherapy combined with chemotherapy is superior to chemotherapy alone as the first-line treatment for advanced biliary tract cancer (median OS: 12.8 months vs. 11.5 months; median PFS: 6.9 months vs. 6.0 months). Subgroup analysis indicated that durvalumab and pembrolizumab had comparable effects (median OS: 12.8 months vs. 12.7 months; median PFS: 7.2 months vs. 6.5 months). We also found that maintaining gemcitabine treatment statistically significantly prolonged the OS but not PFS compared to the gemcitabine limited to 8 cycles treatment. Curiously, gemcitabine maintenance did not numerically improve survival time (OS: 11.5 months vs. 11.5 months; PFS: 5.6 months vs. 6.5 months). In addition to median survival outcomes, survival rates may partially explain the efficacy of gemcitabine maintenance treatment. The 3-year OS rate was 9.3% in the gemcitabine maintenance group compared to 1.3% in the gemcitabine limited-to-8-cycles group.

Due to the survival benefits associated with immunotherapy, our results highlight the contributions of durvalumab and pembrolizumab in advanced biliary tract cancer. However, in the subgroup analysis of durvalumab and pembrolizumab, superiority in OS was observed in the gemcitabine maintenance group compared to the gemcitabine limited-to-8-cycles group. Nonetheless, the median OS was not significantly improved. A retrospective study reported by Jaewon Hyung and colleagues suggested that advanced biliary tract cancer may not benefit from gemcitabine and cisplatin maintenance therapy (21). The median OS was 22.4 months in the maintenance group versus 20.5 months in the observation group (p = 0.162), while the median PFS was 13.2 months in the maintenance group versus 10.4 months in the observation group (p = 0.320). In our data, we observed that patients treated with gemcitabine maintenance had a higher ORR than those who received gemcitabine limited-to-8-cycles (29% vs. 18.7%). Consequently, some patients may not need immunotherapy when receiving gemcitabine maintenance treatment. Additional supporting evidence comes from the reported response rates in the TOPAZ-1 and KEYNOTE-966 trials. In the TOPAZ-1 trial, patients did not receive gemcitabine maintenance treatment, and the objective response rate (ORR) improved with the addition of durvalumab (26% vs. 18.7%) (12). Conversely, in the KEYNOTE-966 trial, patients who received gemcitabine maintenance treatment, and similar ORRs were observed (29% vs. 29%) (14). However, we cannot yet conclude the direct contribution of gemcitabine maintenance therapy. The difference might be attributed to a combination of factors, including tumor type, race, and geographic region. Due to the lack of original response data, the above analysis may indirectly suggest the contributions of immunotherapy and gemcitabine maintenance treatment. Furthermore, in specific circumstances, like high tumor burden, patients might benefit from gemcitabine maintenance therapy.

Identifying the precise population that could benefit from anti-PD-1 and anti-PD-L1 therapies is a challenging process. Before identifying sensitive populations, we recommend determining which patients are unlikely to benefit from immunotherapy. Common characteristics include: female, age < 65, ECOG performance status 0, gallbladder cancer patients, and PD-L1 score < 1 subgroups. Immunotherapy may be less effective in these subgroups. According to the subgroup analyses in the TOPAZ-1 and KEYNOTE-966 trials (12, 14), the population most likely to benefit from immunotherapy appears to be the PD-L1 ≥ 1 subgroup. Additionally, the percentages of patients with PD-L1 ≥ 1 were over 57% in the TOPAZ-1 trial and 68% in the KEYNOTE-966 trial. This information supports our assumption that some populations are suitable for immunotherapy. However, more detailed subgroup analysis, such as microsatellite instability (MSI) status, HBV or HCV status, and tumor mutation burden, is warranted. A genomic analysis conducted by Xu Yang and colleagues confirmed our hypothesis that advanced biliary tract cancer with MSI-H and PD-L1 ≥ 1 was found to be associated with longer OS and PFS (22). However, in both the TOPAZ-1 and KEYNOTE-966 trials, patients with PD-L1 score ≥ 1 demonstrated a borderline improvement in OS in both TOPAZ-1 (HR: 0.79, 95% CI 0.61-1.00) and KEYNOTE-966 trials (HR: 0.84, 95% CI 0.62-1.14), but a significant improvement in PFS in the TOPAZ-1 trial (HR: 0.73, 95% CI 0.59-0.91). Therefore, whether a higher PD-L1 score cut-off is necessary warrants deeper explorations. Additionally, the long-term survivors in the control group of the TOPAZ-1 trial may benefit from the second or later-line immunotherapy (23). Thus, further trials are needed to identify the suitable population of advanced biliary tract cancer for immunotherapy.

Although the cycles of immunotherapy and chemotherapy differed between the TOPAZ-1 and KEYNOTE-966 trials, this heterogeneity prevents us from determining whether durvalumab or pembrolizumab is more suitable for first-line therapy in patients with advanced biliary tract cancer based on our current analysis. Therefore, we suggest that patients with a PD-L1 score ≥ 1 receive durvalumab or pembrolizumab in combination with gemcitabine and cisplatin. Another important issue is whether the increase in survival time by one to two months due to immunotherapy is meaningful for advanced patients. The answer is affirmative. Both trials updated their follow-up data in 2024 (24, 25). A common finding is that the 2-year OS rate was numerically and significantly elevated with durvalumab (23.6% vs. 11.5%) or pembrolizumab (24.6% vs. 19.2%). Based on our reconstructed data, the 2-year OS rate was 24.9% in the immunotherapy plus chemotherapy group and 17.3% in the chemotherapy-alone group (**Table 3**). Combining durvalumab or pembrolizumab with gemcitabine and cisplatin has resulted in survival benefits since the ABC-02 trial. In a recent real-world study by Rimini, patients with advanced biliary tract cancer treated with durvalumab plus gemcitabine and cisplatin achieved a median OS of 15.1 months and a median PFS of 8.2 months (26). The results further support immunotherapy in combination with chemotherapy as a first-line standard of care for advanced biliary tract cancer. This highlights the importance for clinicians to identify the precise population that could benefit from immunotherapy. Additionally, more effective regimens for less responsive populations warrant further exploration.

## Limitations

Several limitations exist in the analysis. All the mimic head-to-head comparisons are indirect. Although there was a low risk of bias among the enrolled trials (Supplement 1), inherent heterogeneities existed between the groups due to the reconstructed patient-level data. Because response data could not be reconstructed, we did not set ORR and DCR as endpoints. Additionally, the safety profiles of durvalumab and pembrolizumab have been confirmed in multiple clinical trials, and both drugs are widely used in real-world clinical practice; therefore, treatment-related adverse events were not included in this analysis.

## Conclusions

Finally, this analysis demonstrates the survival benefits of adding immunotherapy to chemotherapy in patients with advanced biliary tract cancer. Detailed reconstructed survival data can inform future clinical practice. Identifying the appropriate population for immunotherapy requires more prospective clinical trials and real-world studies.

## Data Availability

The raw data supporting the conclusions of this article will be made available by the authors, without undue reservation.
